# Derrisisoflavones H–K and One Isoflavan Derivative from *Derrisrobusta*

**DOI:** 10.1007/s13659-016-0090-x

**Published:** 2016-02-19

**Authors:** Guo-Zhu Wei, Mei-Fen Mao, Xiang-Mei Li, Fu-Cai Ren, Fei Wang

**Affiliations:** BioBioPha Co., Ltd., Kunming, 650201 People’s Republic of China

**Keywords:** *Derris robusta*, Isoflavonoid, Isoflavan, Derrisisoflavone

## Abstract

**Abstract:**

Four hitherto unknown prenylated isoflavonoids, named derrisisoflavones H–K (**1**–**4**) and one new isoflavan, namely 6-hydroxyisosativan (**5**), were isolated from the ethanol extract of *Derrisrobusta*. Their structures were elucidated on the basis of extensive spectroscopic studies. To our knowledge, derrisisoflavones J (**3**) and K (**4**) are the first examples of hydroxyethylated isoflavonoid.

**Graphical Abstract:**

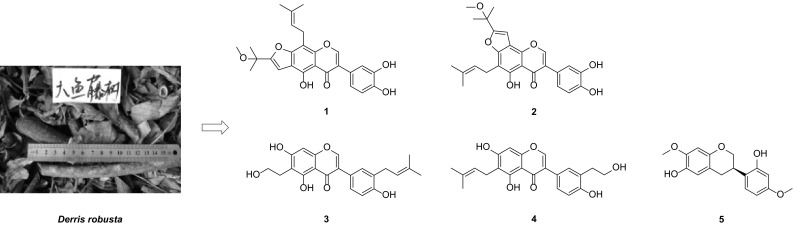

**Electronic supplementary material:**

The online version of this article (doi:10.1007/s13659-016-0090-x) contains supplementary material, which is available to authorized users.

## Introduction

**Fig. 1:**
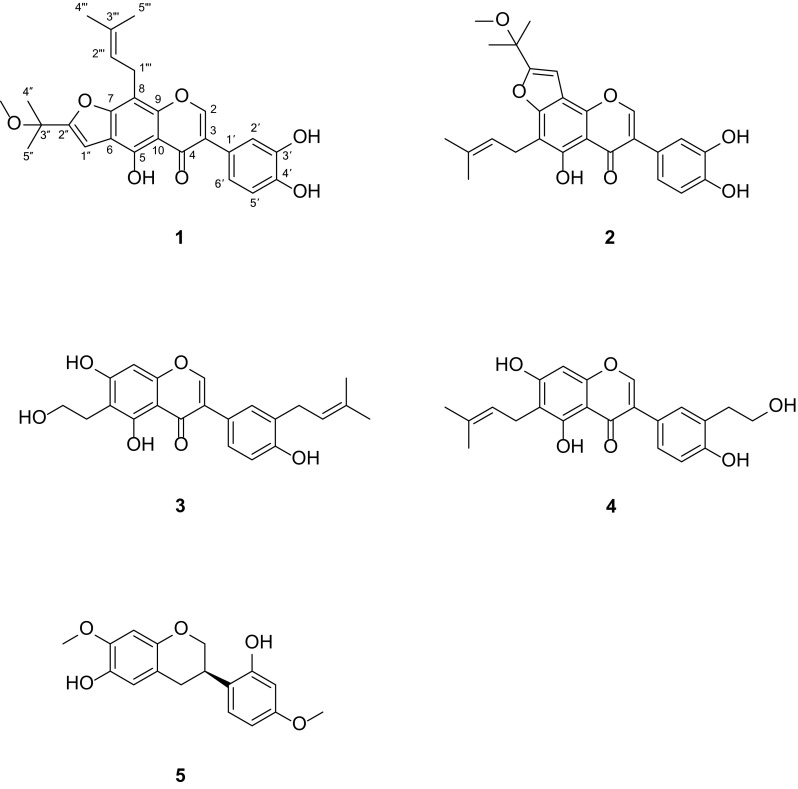
Structures of compounds **1**–**5**

*Derris* is a genus belonging to the Leguminosae family with about 800 species that are widely distributed in tropical, subtropical areas of Asia and Africa [1]. Published studies have shown that the genus is a rich source of pterocarpans, flavonoids, particularly prenylated isoflavonoids and flavonoids [2–5] and these phytochemicals are associated with a broad spectrum of biological activities, including insecticidal, antimicrobial, cytotoxic, and antioxidant activities [3–7]. As part of a BioBioPha [http://www.chemlib.cn/] objective to assemble a large-scale natural product library valuable in the discovery of new drug leads from nature [8–10], the phytochemical investigation on the twigs and leaves of *Derrisrobusta* led to the isolation of four new prenylated isoflavonoids, named derrisisoflavones H–K (**1**–**4**), and a new isoflavan, namely 6-hydroxyisosativan (**5**). This paper describes the isolation and structural elucidation of five new compounds (Fig. 1).

## Results and Discussion

**Fig. 2:**
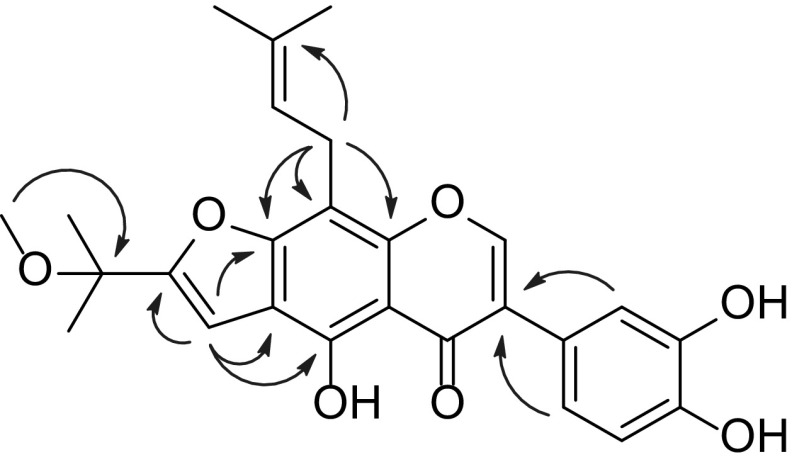
Selected HMBC () correlations of derrisisoflavone H (**1**)

Compound **1** was obtained as a yellow amorphous powder and had a molecular formula C_26_H_26_O_7_ determined by its HRESIMS, showing a negative molecular ion peak at *m*/*z* 449.1608 [M − H]^−^ (calcd. for C_26_H_25_O_7_, 449.1606). The UV spectrum of **1** with a set of absorption maxima at 268, 297 (sh), 359 nm suggested that it had an isoflavone skeleton as the chromophore [11]. This inference was further supported by characteristic proton singlet at *δ*_H_ 8.17 (H-2) and *sp*^2^ methine carbon at *δ*_C_ 155.6 (C-2). The ^1^H NMR spectrum (Table 1) showed a set of signals at *δ*_H_ 7.05 (br. s), 6.87 (br. d, *J* = 8.0 Hz) and 6.82 (d, *J* = 8.0 Hz) due to a 1,3,4-trisubstituted benzene ring, two aromatic or olefinic protons at *δ*_H_ 6.86 (s) and 5.30 (t, *J* = 6.8 Hz), one methylene signal at *δ*_H_ 3.64 (d, *J* = 6.8 Hz), and five methyl singlets at *δ*_H_ 3.11, 1.85, 1.67, 1.60 and 1.60. The ^13^C NMR (DEPT) spectrum (Table 2) displayed a total of 26 carbon resonances, including five methyls, one methylene, six methines and 14 quaternary carbons. The above NMR spectroscopic features were very similar to those of 5,4′-dihydroxy-8-(3,3-dimethylallyl)-2″-methoxyisopropylfurano[4,5:6,7]isoflavone [12], and the most dramatic difference was the presence of an additional hydroxy group in **1**. The hydroxy group was located at C-3′, on the basis of the HMBC correlations from the protons at *δ*_H_ 7.05 (br. s, H-2′) and 6.87 (br. d, *J* = 8.0 Hz, H-6′) to the carbon at *δ*_C_ 123.7 (s, C-3) (Fig. 2). The HMBC correlations from the protons at *δ*_H_ 3.64 (2H, d, *J* = 6.8 Hz, H-1′′′) to the carbons at *δ*_C_ 158.6 (s, C-7), 105.2 (s, C-8), and 152.4 (s, C-9) verified the location of the prenyl group [*δ*_H_ 1.67, 1.85 (each s), 3.64 (d, *J* = 6.8 Hz), and 5.30 (t, *J* = 6.8 Hz)] at C-8. Furthermore, the correlations from the proton at *δ*_H_ 6.86 (s, H-1″) to the carbons at *δ*_C_ 154.2 (s, C-5), 114.1 (s, C-6), and 158.6 (s, C-7) confirmed that the furan ring, derived from a prenyl group, was fused along the C-6 to C-7 bond. Consequently, the structure of **1** was determined and named as derrisisoflavone H.

**Table 1:** ^1^H NMR spectroscopic data for derrisisoflavones H–K (**1**–**4**) and 6-hydroxyisosativan (**5**)

No.	**1** ^a^	**2** ^b^	**3** ^a^	**4** ^a^	**5** ^a^
2	8.17 (s)	8.51 (s)	7.97 (s)	8.00 (s)	4.17 (ddd, 10.2, 3.3, 1.9, H_*eq*_)
					3.90 (t, 10.2, H_*ax*_)
3					3.43 (dddd, 10.7, 10.2, 5.3, 3.3, H_*ax*_)
4					2.91 (dd, 15.9, 10.7, H_*ax*_)
					2.73 (ddd, 15.9, 5.3, 1.9, H_*eq*_)
5					6.51 (s)
8			6.37 (s)	6.36 (s)	6.37 (s)
2′	7.05 (br. s)	7.05 (d, 1.6)	7.19 (d, 2.0)	7.25 (d, 2.3)	
3′					6.38 (d, 2.5)
5′	6.82 (d, 8.0)	6.81 (d, 8.0)	6.79 (d, 8.2)	6.82 (d, 8.2)	6.36 (dd, 8.4, 2.5)
6′	6.87 (br. d, 8.0)	6.85 (dd, 8.0, 1.6)	7.15 (dd, 8.2, 2.0)	7.21 (dd, 8.2, 2.3)	6.95 (d, 8.4)
1″	6.86 (s)	3.48 (d, 7.3)	2.92 (t, 7.3)	3.30 (overlapped)	
2″		5.29 (t, 7.3)	3.68 (t, 7.3)	5.22 (t, 7.2)	
4″	1.60 (s)	1.62 (s)		1.65 (s)	
5″	1.60 (s)	1.79 (s)		1.77 (s)	
1′′′	3.64 (d, 6.8)	7.07 (s)	3.32 (d, 7.3)	2.87 (t, 7.0)	
2′′′	5.30 (t, 6.8)		5.33 (t, 7.3)	3.78 (t, 7.0)	
4′′′	1.67 (s)	1.56 (s)	1.72 (s)		
5′′′	1.85 (s)	1.56 (s)	1.72 (s)		
5-OH		13.21 (s)			
3′-OH		9.14 (br. s)			
4′-OH		9.14 (br. s)			
7-OCH_3_					3.78 (s)
4′-OCH_3_					3.71 (s)
3″-OCH_3_	3.11 (s)				
3′′′-OCH_3_		3.00 (s)			

^a^Measured in CD_3_OD (*δ*_H_ 3.30 ppm)

^b^Measured in DMSO-*d*_6_ (*δ*_H_ 2.50 ppm)

**Table 2:** ^13^C NMR spectroscopic data for derrisisoflavones H–K (**1**–**4**) and 6-hydroxyisosativan (**5**)

No.	**1** ^a^	**2** ^b^	**3** ^a^	**4** ^a^	**5** ^a^
2	155.6 (d)	154.1 (d)	154.5 (d)	154.6 (d)	71.0 (t)
3	123.7 (s)	123.5 (s)	125.0 (s)	124.7 (s)	33.1 (d)
4	184.2 (s)	181.4 (s)	182.4 (s)	182.3 (s)	31.4 (t)
5	154.2 (s)	154.6 (s)	161.3 (s)	160.5 (s)	116.4 (d)
6	114.1 (s)	107.6 (s)	109.9 (s)	113.1 (s)	141.0 (s)
7	158.6 (s)	157.0 (s)	164.2 (s)	163.7 (s)	148.1 (s)
8	105.2 (s)	107.9 (s)	94.2 (d)	93.9 (d)	101.4 (d)
9	152.4 (s)	147.7 (s)	157.9 (s)	157.6 (s)	148.8 (s)
10	107.6 (s)	107.4 (s)	106.2 (s)	106.1 (s)	115.0 (s)
1′	123.7 (s)	121.5 (s)	123.4 (s)	123.4 (s)	121.4 (s)
2′	117.5 (d)	116.8 (d)	131.4 (d)	132.8 (d)	157.2 (s)
3′	146.2 (s)	145.0 (s)	129.5 (s)	126.7 (s)	102.3 (d)
4′	146.8 (s)	145.8 (s)	156.5 (s)	157.0 (s)	160.8 (s)
5′	116.3 (d)	115.5 (d)	115.8 (d)	116.1 (d)	105.6 (d)
6′	121.8 (d)	120.3 (d)	128.7 (d)	129.5 (d)	128.8 (d)
1″	102.7 (d)	21.6 (t)	26.8 (t)	22.3 (t)	
2″	161.3 (s)	120.9 (d)	61.9 (t)	123.4 (d)	
3″	74.7 (s)	132.1 (s)		132.1 (s)	
4″	25.5 (q)	25.5 (q)		26.0 (q)	
5″	25.5 (q)	17.6 (q)		17.9 (q)	
1′′′	22.9 (t)	101.2 (d)	29.3 (t)	35.1 (t)	
2′′′	122.2 (d)	159.3 (s)	123.9 (d)	63.0 (t)	
3′′′	133.8 (s)	72.8 (s)	133.1 (s)		
4′′′	25.9 (q)	24.9 (q)	25.9 (q)		
5′′′	18.0 (q)	24.9 (q)	17.9 (q)		
7-OCH_3_					56.3 (q)
4′-OCH_3_					55.5 (q)
3″-OCH_3_	51.4 (q)				
3′′′-OCH_3_		50.4 (q)			

^a^Measured in CD_3_OD (*δ*_C_ 49.0 ppm)

^b^Measured in DMSO-*d*_6_ (*δ*_C_ 39.5 ppm)

Compound **2**, was purified as a yellow amorphous powder and had the same molecular formula as **1** based on its HRESIMS (neg.): *m*/*z* 449.1607 [M − H]^−^ (calcd. for C_26_H_25_O_7_, 449.1606). The NMR spectroscopic data of **2** (Tables 1, 2) were very similar to those of **1**. The structural discrepancy was only from the switch positions of prenyl group and furan ring on ring A. The positions were verified by the HMBC correlations from the protons at *δ*_H_ 3.48 (2H, d, *J* = 7.3 Hz, H-1″) and 13.21 (s, 5-OH) to the carbon at *δ*_C_ 154.6 (s, C-5), and from the protons at *δ*_H_ 7.07 (s, H-1′′′) and 8.51 (s, H-2) to the carbon at *δ*_C_ 147.7 (s, C-9), respectively. Accordingly, the structure of **2** was elucidated as shown and given the name derrisisoflavone I.

Compound **3**, was isolated as a white amorphous powder, with a molecular formula of C_22_H_22_O_6_ according to its HRESIMS (pos.): *m*/*z* 405.1305 [M + Na]^+^ (calcd. for C_22_H_22_O_6_Na, 405.1309). The general features of NMR spectra (Tables 1, 2) of **3** were similar to those of lupalbigenin, a diprenylated isoflavone [13], except for the signals of a hydroxyethyl moiety [*δ*_H_ 2.92 (t, *J* = 7.3 Hz), 3.68 (t, *J* = 7.3 Hz); *δ*_C_ 26.8 (t), 61.9 (t)] instead of one of prenyl group in the latter. The hydroxyethyl group was linked to C-6 on the basis of the following HMBC correlations: from the protons at *δ*_H_ 2.92 (t, *J* = 7.3 Hz, H-1″) to the carbons at *δ*_C_ 161.3 (s, C-5), 109.9 (s, C-6) and 164.2 (s, C-7), and from the protons at *δ*_H_ 7.97 (s, H-2) and 6.37 (s, H-8) to the carbon at *δ*_C_ 157.9 (s, C-9). Similarly, the connection of the prenyl group to C-3′ was established by the correlation from the proton at *δ*_H_ 7.19 (d, *J* = 2.0 Hz, H-2′) to the carbon at 29.3 (t, C-1′′′). Therefore, the structure of **3** was characterized and named as derrisisoflavone J.

Compound **4** was afforded as a white amorphous powder and possessed the same molecular formula as **3** according to its HRESIMS (pos.): *m*/*z* 405.1307 [M + Na]^+^ (calcd. for C_22_H_22_O_6_Na, 405.1309). The NMR data (Tables 1, 2) were very similar to those of **3**, which allowed us to infer that their structural discrepancy may result from the different substitution patterns of the hydroxyethyl and prenyl groups. This deduction was confirmed by the HMBC correlations from the protons at 3.30 (overlapped, H-1″) to *δ*_C_ 160.5 (s, C-5), 113.1 (s, C-6) and 163.7 (s, C-7), and from the proton at *δ*_H_ 7.25 (d, *J* = 2.3 Hz, H-2′) to *δ*_C_ 35.1 (t, C-1′′′). Therefore, the structure of **4** was established as shown and given the name derrisisoflavone K.

Compound **5**, a white amorphous powder, had a molecular formula of C_17_H_18_O_5_ by its HRESIMS (pos.): *m*/*z* 325.1031 [M + Na]^+^ (calcd. for C_17_H_18_O_5_Na, 325.1046). Its ^1^H NMR spectrum (Table 1) displayed an ABX-type aromatic proton system [*δ*_H_ 6.38 (d, *J* = 2.5 Hz), 6.36 (dd, *J* = 8.4, 2.5 Hz), and 6.95 (d, *J* = 8.4 Hz)], two aromatic singlets at *δ*_H_ 6.51 and 6.37, two methoxy signals at *δ*_H_ 3.78 and 3.71, and a set of signals [*δ*_H_ 4.17 (ddd, *J* = 10.2, 3.3, 1.9 Hz, H_*eq*_-2), 3.90 (t, *J* = 10.2 Hz, H_*ax*_-2), 3.43 (dddd, *J* = 10.7, 10.2, 5.3, 3.3 Hz, H_*ax*_-3), 2.91 (dd, *J* = 15.9, 10.7 Hz, H_*ax*_-4), and 2.73 (ddd, *J* = 15.9, 5.3, 1.9 Hz, H_*eq*_-4)] due to ring C protons of an isoflavan. The above NMR signals were similar to those of isosativan (also called 7-*O*-methylvestitol) [14], and a prominent difference was two aromatic singlets at *δ*_H_ 6.51 and 6.37 instead of one of ABX-type system of isosativan. By careful analysis of the MS and NMR data, the isoflavan was inferred as a hydroxylated derivative of isosativan. The additional hydroxy group was located at C-6 by the HMBC correlations from the proton singlet at *δ*_H_ 6.51 (H-5) to the carbon at *δ*_C_ 31.4 (t, C-4), and from the methoxy signal at *δ*_H_ 3.78 (7-OMe) to the carbons at *δ*_C_ 148.1 (s, C-7) and 101.4 (d, C-8). Thereupon, the structure of **5** was established and named 6-hydroxyisosativan. The absolute configuration at C-3 was postulated as being *R*-form in the light of a negative specific rotation value (−11.7, MeOH), consistent with those of (3*R*)-vestitol derivatives [15].

## Experimental Section

### General Experimental Procedures

Optical rotation was measured on a Jasco P-1020 automatic digital polarimeter. UV data were obtained from HPLC online analysis. NMR spectra were carried out on a Bruker AV-400, Bruker DRX-500 or Bruker AV-600 instrument with deuterated solvent signals used as internal standards. ESI and HRESIMS were performed with a Shimadzu LC-IT-TOF mass spectrometer equipped with an ESI interface (Shimadzu, Kyoto, Japan). Silica gel 200–300 mesh (Qingdao Marine Chemical Inc., Qingdao, China), Chromatorex C-18 (40–75 μm, Fuji Silysia Chemical Ltd., Japan) and Sephadex LH-20 (Amersham Biosciences, Uppsala, Sweden) were used for normal pressure column chromatography (CC). Fractions were monitored and analyzed by TLC, in combination with Agilent 1200 series HPLC system equipped by Extend-C18 column (5 μm, 4.6 × 150 mm).

### Plant Material

The twigs and leaves of *D*. *robusta* were collected from the Pu’er region of Yunnan Province, People’s Republic of China, in May 2011, and identified by Mr. Yu Chen of Kunming Institute of Botany, Chinese Academy of Sciences. A voucher specimen (BBP0350022DR) was deposited at BioBioPha Co., Ltd.

### Extraction and Isolation

The air-dried and powdered twigs and leaves (12.0 kg) of *D*. *robusta* were extracted with EtOH-H_2_O (95:5, v/v; 3 × 20 L, each 4 days) at room temperature, and the combined filtrates were concentrated under reduced pressure to give crude extract (ca. 870 g), which was further fractionated by silica gel CC successively eluted with a gradient of increasing acetone in petroleum ether (PE) (10:1 → 0:1, v/v) and then MeOH to obtain nine fractions A–I. Fraction D (PE/acetone, 5:1, v/v) was subjected to silica gel CC (CHCl_3_/MeOH, 100:0 → 100:1, v/v) and Sephadex LH-20 (CHCl_3_/MeOH, 1:1, v/v) to give **5** (14 mg). Fraction E (PE/acetone, 4:1, v/v) was isolated on silica gel CC (CHCl_3_/MeOH, 100:1 → 5:1, v/v), RP-18 (30 % MeOH/H_2_O, v/v), and Sephadex LH-20 (MeOH) to yield **1** (7 mg), **2** (11 mg), and **4** (14 mg). Fraction H (PE/acetone, 1:1) was purified by silica gel CC (CHCl_3_/MeOH, 10:1 → 2:1, v/v) and repeated Sephadex LH-20 (MeOH) to afford **3** (10 mg). The retention times (*t*_R_) of **1**–**5** on an analytical HPLC Extend-C18 column (20 % → 100 % MeOH in H_2_O over 8.0 min followed by 100 % MeOH to 13.0 min, 1.0 ml/min, 25 °C) were 10.32, 10.12, 9.13, 9.16, and 7.76 min, respectively.

### Derrisisoflavone H (**1**)

Yellow amorphous powder; UV (MeOH) *λ*_max_ (log *ε*): 268 (4.75), 297 (sh) (4.23), 359 (3.60) nm; ^1^H NMR data: see Table 1; ^13^C NMR data: see Table 2; ESIMS (neg.): *m/z* 449 [M − H]^−^; HRESIMS (neg.): 449.1608 [M − H]^−^ (calcd. for C_26_H_25_O_7_, 449.1606).

### Derrisisoflavone I (**2**)

Yellow amorphous powder; UV (MeOH) *λ*_max_ (log *ε*): 263 (4.75), 302 (sh) (4.35), 357 (sh) (3.68) nm; ^1^H NMR data: see Table 1; ^13^C NMR data: see Table 2; ESIMS (neg.): *m/z* 449 [M − H]^−^; HRESIMS (neg.): *m/z* 449.1607 [M − H]^−^ (calcd. for C_26_H_25_O_7_, 449.1606).

### Derrisisoflavone J (**3**)

White amorphous powder; UV (MeOH) *λ*_max_ (log *ε*): 213 (4.59), 267 (4.56), 338 (sh) (3.59) nm; ^1^H NMR data: see Table 1; ^13^C NMR data: see Table 2; ESIMS (pos.): *m/z* 405 [M + Na]^+^; HRESIMS (pos.): *m*/*z* 405.1305 [M + Na]^+^ (calcd. for C_22_H_22_O_6_Na, 405.1309).

### Derrisisoflavone K (**4**)

White amorphous powder; UV (MeOH) *λ*_max_ (log *ε*): 214 (4.59), 268 (4.57), 336 (sh) (3.67) nm; ^1^H NMR data: see Table 1; ^13^C NMR data: see Table 2; ESIMS (pos.): *m/z* 405 [M + Na]^+^; HRESIMS (pos.): *m*/*z* 405.1307 [M + Na]^+^ (calcd. for C_22_H_22_O_6_Na, 405.1309).

### 6-Hydroxyisosativan (**5**)

White amorphous powder; UV (MeOH) *λ*_max_ (log *ε*): 226 (sh) (4.22), 287 (3.89) nm; $\left[ \alpha \right]_{\text{D}}^{23}$ −11.7 (*c* 0.2, MeOH); ^1^H NMR data: see Table 1; ^13^C NMR data: see Table 2; ESIMS (pos.): *m/z* 325 [M + Na]^+^; HRESIMS (pos.): *m*/*z* 325.1031 [M + Na]^+^ (calcd. for C_17_H_18_O_5_Na, 325.1046).

## Electronic supplementary material

Supplementary material 1 (DOCX 1082 kb)
