# Supplementary material for: Derrisisoflavones H–K and One Isoflavan Derivative from Derrisrobusta
Source: Nat Prod Bioprospect. 2016 Feb 19;6(2):111–6. doi: 10.1007/s13659-016-0090-x (PMC4805655; doi:10.1007/s13659-016-0090-x)

**Electronic Supplementary Material**

**Derrisisoflavones H–K and one isoflavan derivative from** ***Derris* *robusta***

Guo-Zhu Wei • Mei-Fen Mao • Xiang-Mei Li • Fu-Cai Ren • Fei Wang

BioBioPha Co., Ltd., Kunming 650201, People’s Republic of China

*To whom correspondence should be addressed.

E-mail: f.wang@mail.biobiopha.com

**Content list:**

**S1.** ^1^H NMR spectrum (400 MHz, CD_3_OD) of derrisisoflavone H (**1**).

**S2.** ^13^C NMR spectrum (100 MHz, CD_3_OD) of derrisisoflavone H (**1**).

**S3.** HMBC spectrum (500 MHz, CD_3_OD) of derrisisoflavone H (**1**).

**S4.** ^1^H NMR spectrum (400 MHz, DMSO-*d*_6_) of derrisisoflavone I (**2**).

**S5.** ^13^C NMR spectrum (100 MHz, DMSO-*d*_6_) of derrisisoflavone I (**2**).

**S6.** HMBC spectrum (500 MHz, DMSO-*d*_6_) of derrisisoflavone I (**2**).

**S7.** ^1^H NMR spectrum (400 MHz, CD_3_OD) of derrisisoflavone J (**3**).

**S8.** ^13^C NMR spectrum (100 MHz, CD_3_OD) of derrisisoflavone J (**3**).

**S9.** HMBC spectrum (500 MHz, CD_3_OD) of derrisisoflavone J (**3**).

**S10.** ^1^H NMR spectrum (400 MHz, CD_3_OD) of derrisisoflavone K (**4**).

**S11.** ^13^C NMR spectrum (100 MHz, CD_3_OD) of derrisisoflavone K (**4**).

**S12.** HMBC spectrum (500 MHz, CD_3_OD) of derrisisoflavone K (**4**).

**S13.** ^1^H NMR spectrum (400 MHz, CD_3_OD) of 6-hydroxyisosativan (**5**).

**S14.** ^13^C NMR spectrum (100 MHz, CD_3_OD) of 6-hydroxyisosativan (**5**).

**S15.** HMBC spectrum (600 MHz, CD_3_OD) of 6-hydroxyisosativan (**5**).

**S1.** ^1^H NMR spectrum (400 MHz, CD_3_OD) of derrisisoflavone H (**1**).


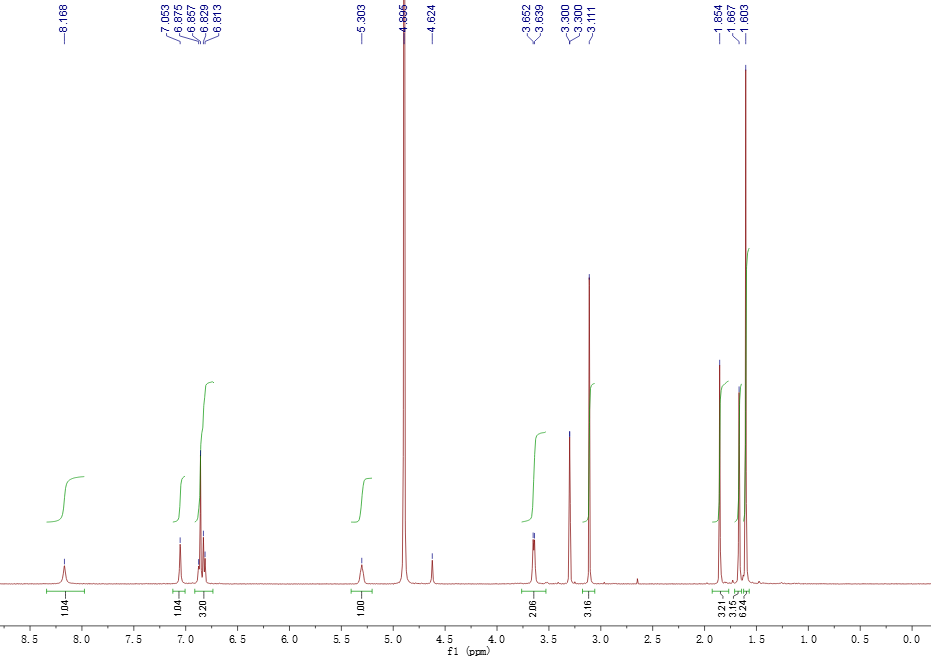

**S2.** ^13^C NMR spectrum (100 MHz, CD_3_OD) of derrisisoflavone H (**1**).


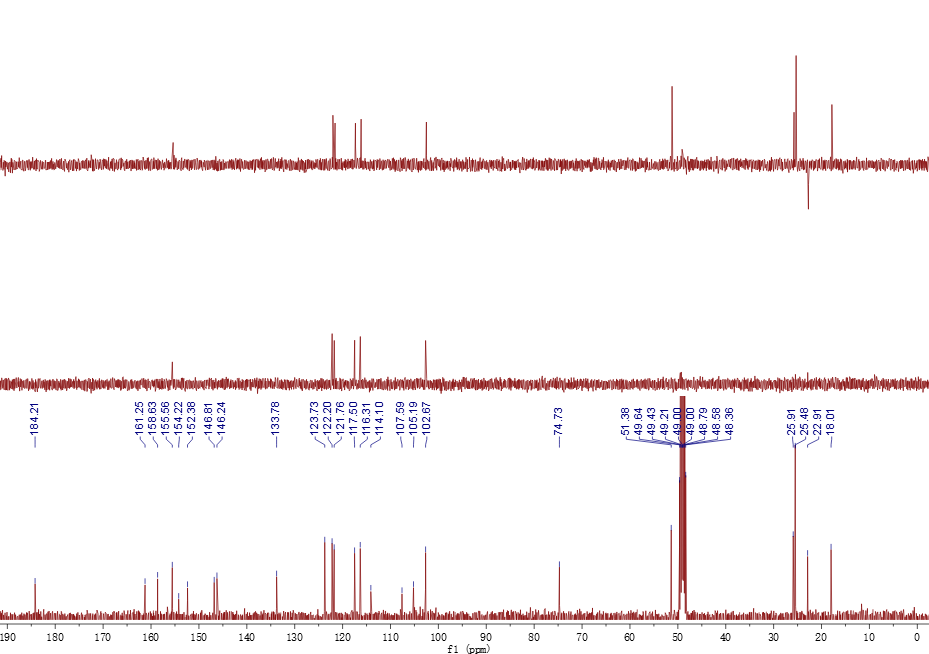

**S3.** HMBC spectrum (500 MHz, CD_3_OD) of derrisisoflavone H (**1**).


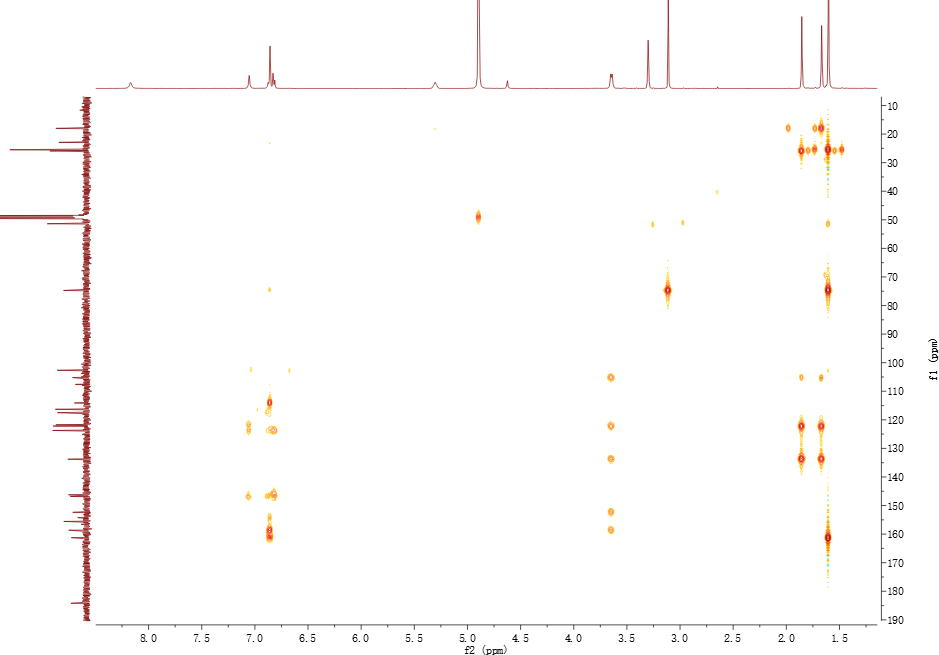

**S4.** ^1^H NMR spectrum (400 MHz, DMSO-*d*_6_) of derrisisoflavone I (**2**).


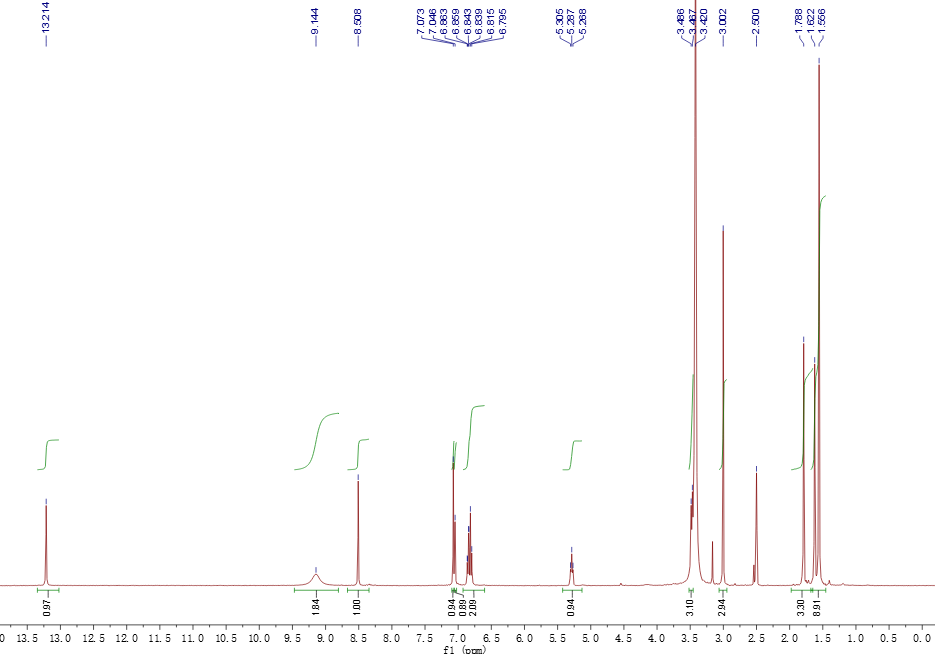

**S5.** ^13^C NMR spectrum (100 MHz, DMSO-*d*_6_) of derrisisoflavone I (**2**).


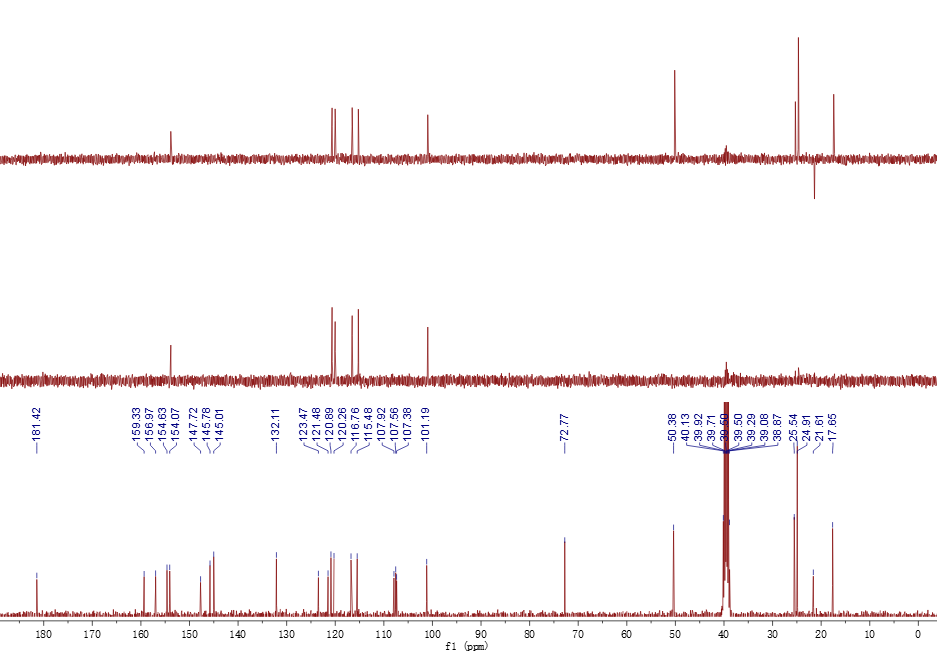

**S6.** HMBC spectrum (500 MHz, DMSO-*d*_6_) of derrisisoflavone I (**2**).


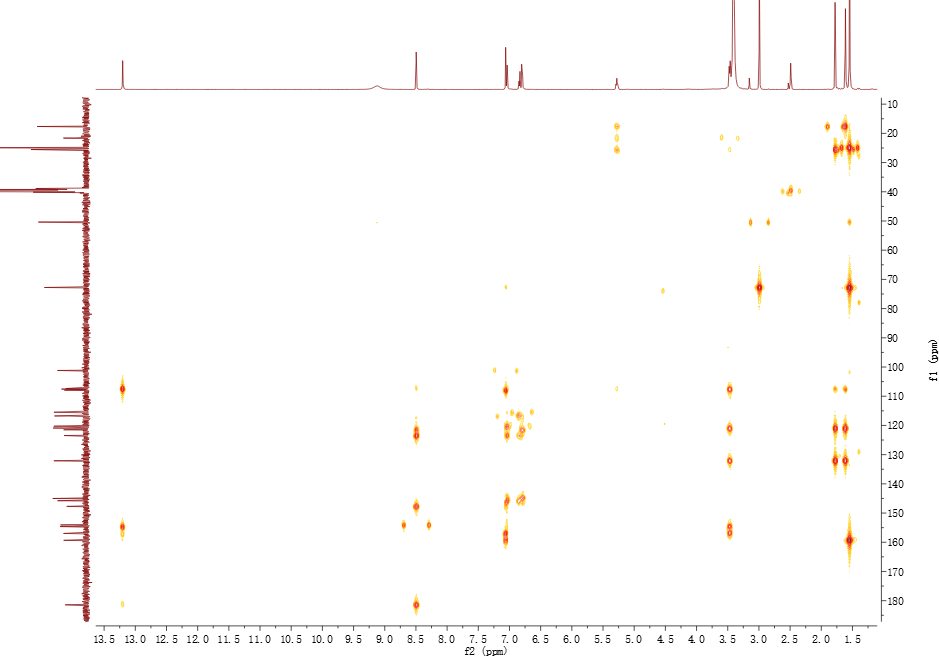

**S7.** ^1^H NMR spectrum (400 MHz, CD_3_OD) of derrisisoflavone J (**3**).


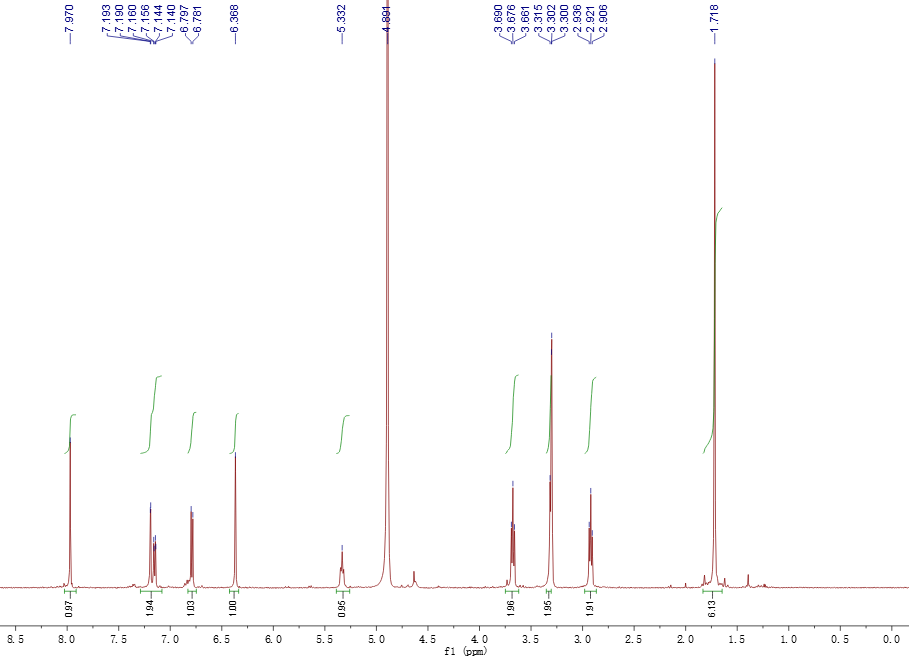

**S8.** ^13^C NMR spectrum (100 MHz, CD_3_OD) of derrisisoflavone J (**3**).


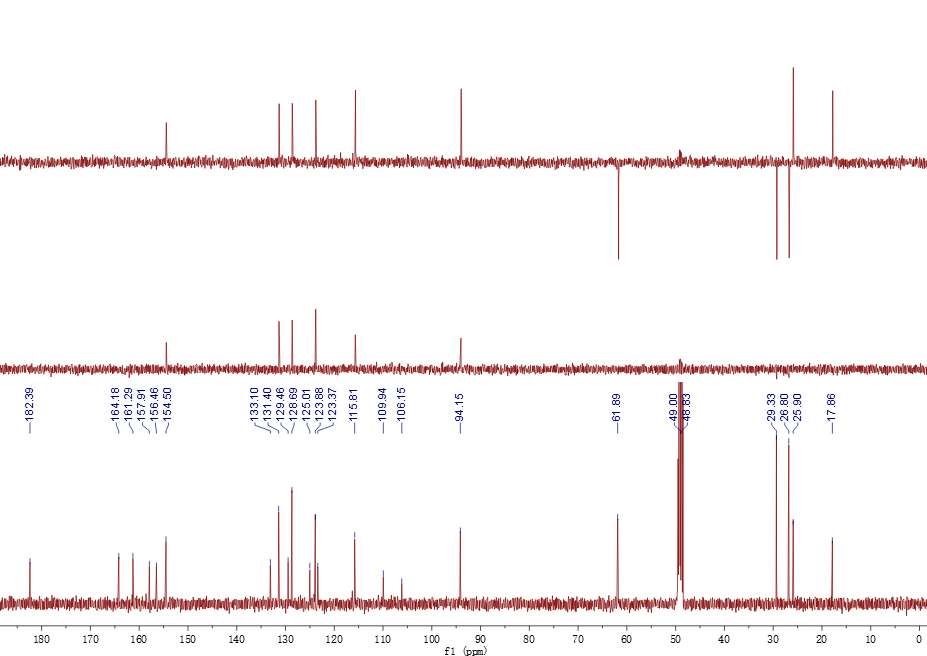

**S9.** HMBC spectrum (500 MHz, CD_3_OD) of derrisisoflavone J (**3**).


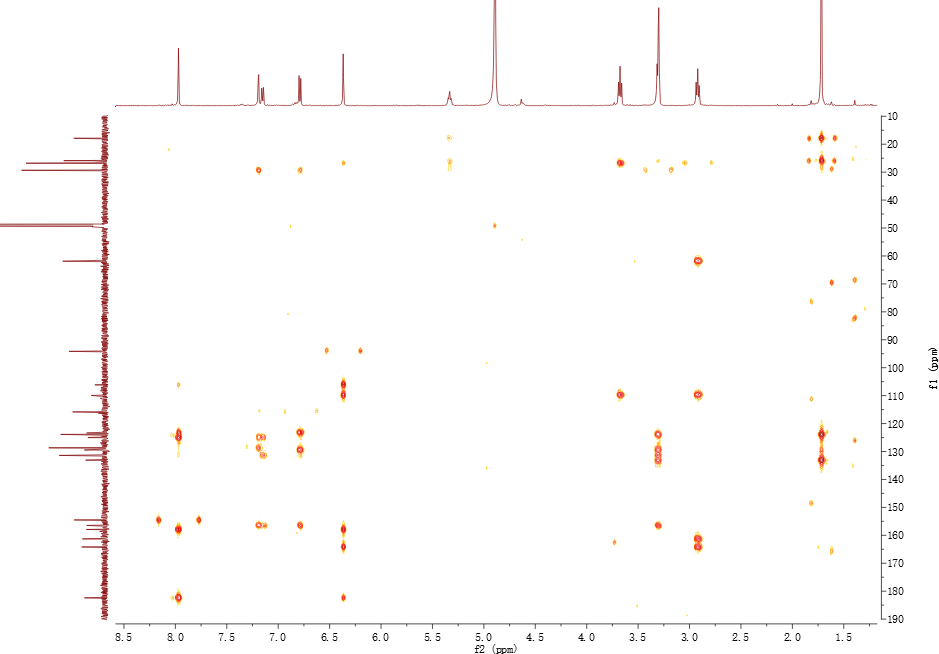

**S10.** ^1^H NMR spectrum (400 MHz, CD_3_OD) of derrisisoflavone K (**4**).


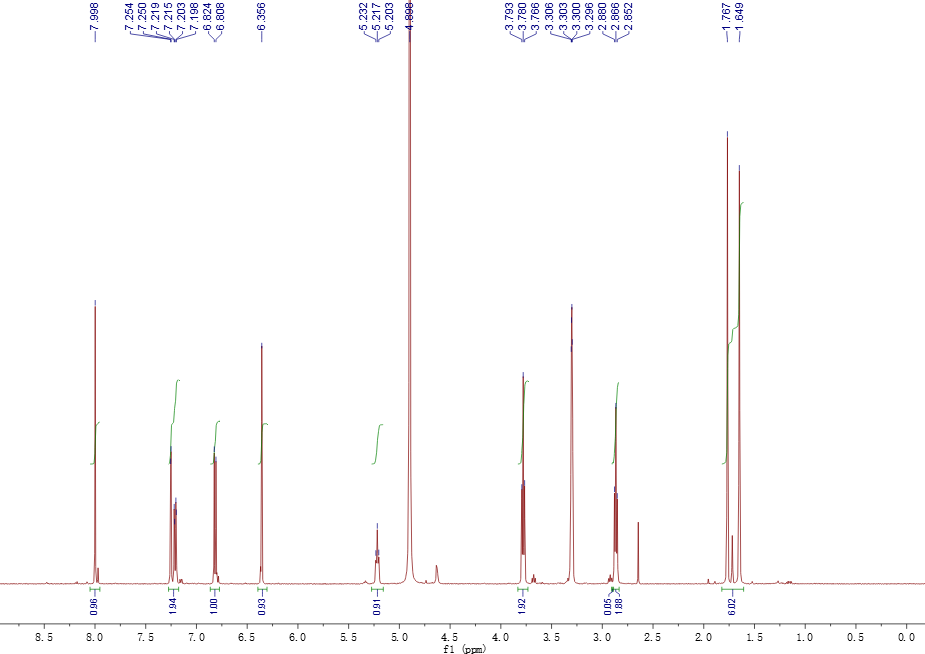

**S11.** ^13^C NMR spectrum (100 MHz, CD_3_OD) of derrisisoflavone K (**4**).


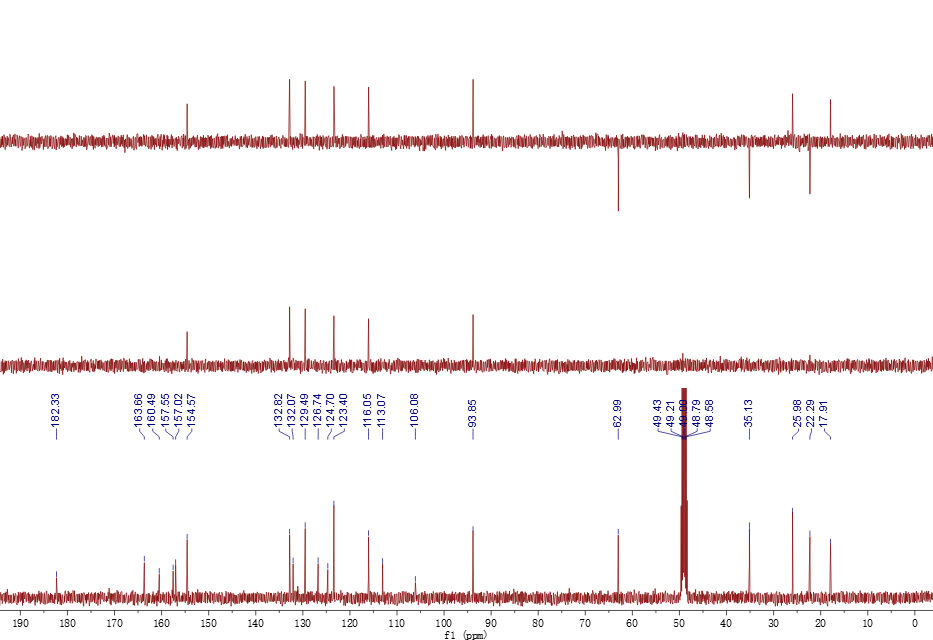

**S12.** HMBC spectrum (500 MHz, CD_3_OD) of derrisisoflavone K (**4**).


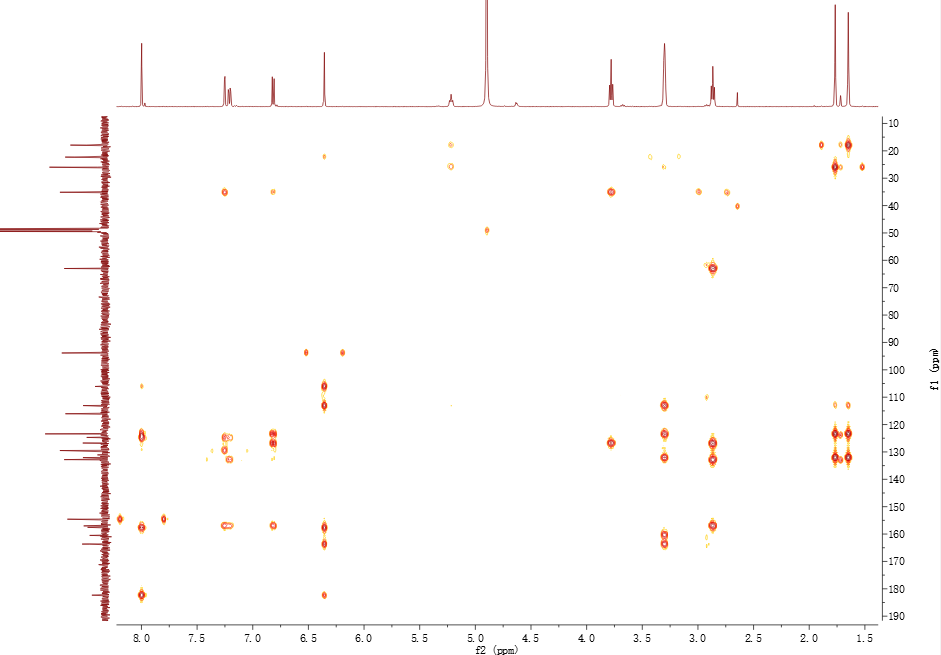

**S13.** ^1^H NMR spectrum (400 MHz, CD_3_OD) of 6-hydroxyisosativan (**5**).


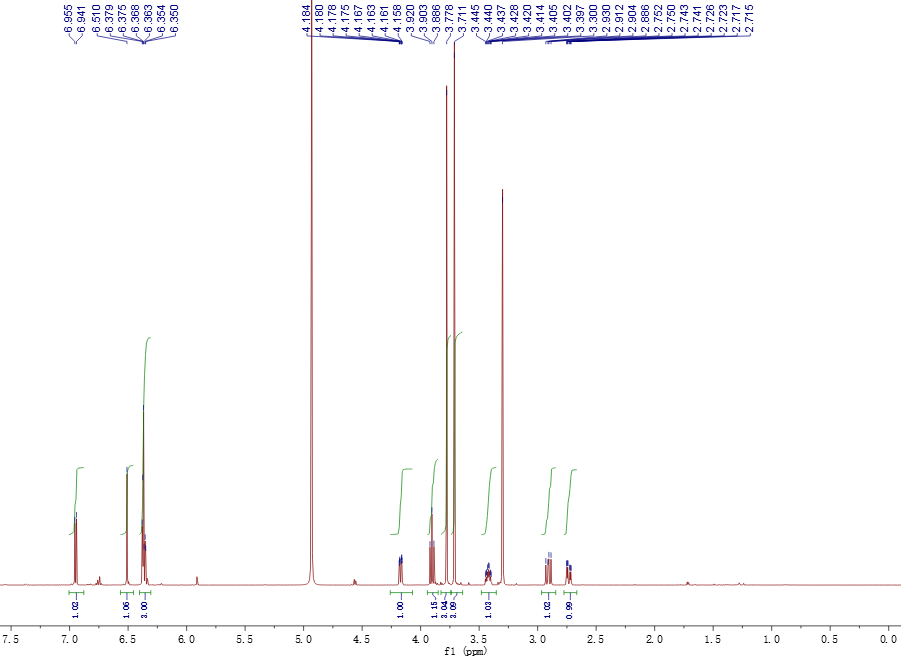

**S14.** ^13^C NMR spectrum (100 MHz, CD_3_OD) of 6-hydroxyisosativan (**5**).


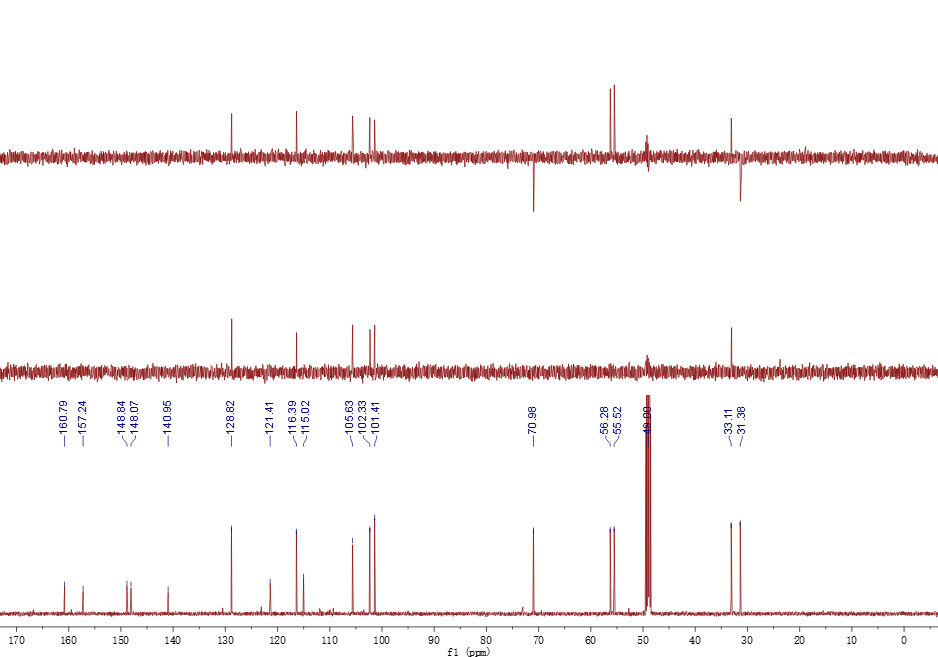

**S15.** HMBC spectrum (600 MHz, CD_3_OD) of 6-hydroxyisosativan (**5**).


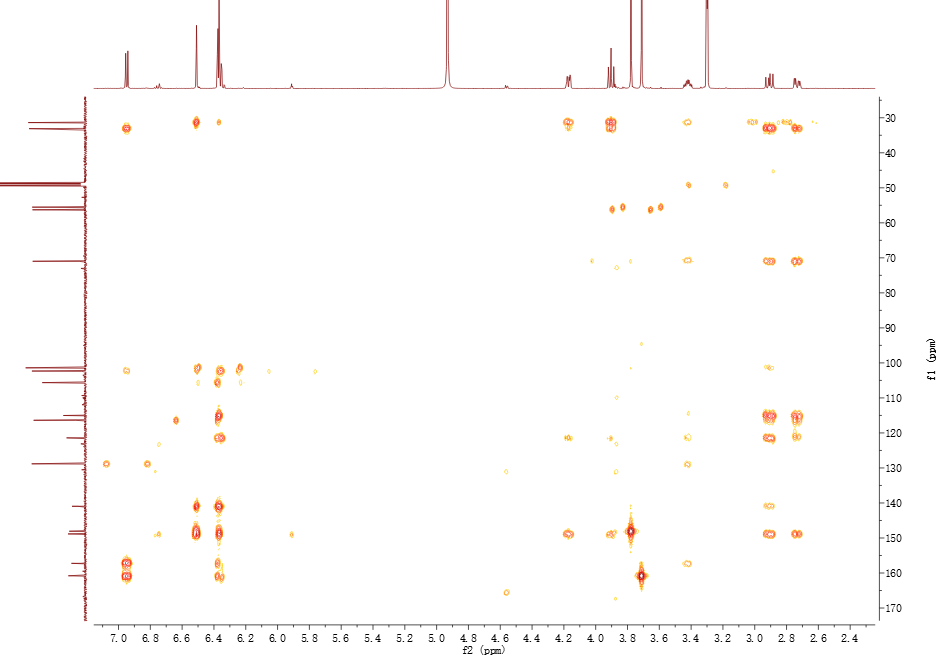

Supplement: Supplementary file 1 — Supplementary material 1 (DOCX 1082 kb) [file 13659_2016_90_MOESM1_ESM.docx]
